# Can Environmental Quality Improvement and Emission Reduction Targets Be Realized Simultaneously? Evidence from China and A Geographically and Temporally Weighted Regression Model

**DOI:** 10.3390/ijerph15112343

**Published:** 2018-10-24

**Authors:** Feng Dong, Yue Wang, Xiaojie Zhang

**Affiliations:** School of Management, China University of Mining and Technology, Xuzhou 221116, China; m18234567643@163.com (Y.W.); 15735172611@163.com (X.Z.)

**Keywords:** industrial pollution emissions, vertical and horizontal scatter degree method, carbon density, GTWR

## Abstract

The reductions of industrial pollution and greenhouse gas emissions are important actions to create an ecologically stable civilization. However, there are few reports on the interaction and variation between them. In this study, the vertical and horizontal scatter degree method is used to calculate a comprehensive index of industrial pollution emissions. Then based on carbon density, a geographically and temporally weighted regression (GTWR) model is developed to examine the interaction between industrial pollution emissions and carbon emissions. The results specify that there exists spatial autocorrelation for carbon density in China. Overall, the average effect of industrial pollution emissions on carbon density is positive. This indicates that industrial pollution emissions play a driving role in carbon density on the whole, while there are temporal and spatial differences in the interactions at the provincial level. According to the Herfindahl index, neither time nor space can be neglected. Moreover, according to the traditional division of eastern, central and western regions in China, the situation in 30 provinces is examined. Results show that there is little difference in the parameter-estimated results between neighboring provinces. In many provinces, the pull effect of industrial pollution emissions on carbon density is widespread. Thus, carbon emissions could be reduced by controlling industrial pollution emissions in more than 60% of regions. In a few other regions, such as Shanghai and Heilongjiang, the industrial pollution emissions do not have a pull effect on carbon density. But due to spatial and temporal heterogeneity, the effects are different in different regions at different times. It is necessary to consider the reasons for the changes combined with other factors. Finally, the empirical results support pertinent suggestions for controlling future emissions, such as optimizing energy mix and reinforcing government regulation.

## 1. Introduction

Carbon emissions have been a topical issue within the international community [1]. China is the world’s largest energy consumer and it has been the world’s largest carbon emitter [2]. In 2007, China’s carbon emissions exceeded those of the United States of America [3]. In the 21st century, the growth of global carbon emission intensity has been mainly driven by China [4]. Under the pressure of international emission reduction targets and the need to improve domestic environmental quality, a series of emission reduction commitments have been put forward by China’s national government. For example, compared with that in 2005, the carbon emission intensity in 2020 and 2030 will be lowered by 40–45% and 60–65% [5], respectively. Furthermore, the peak of carbon emissions will occur around 2030 [6]. In order to achieve carbon reduction targets, the Chinese government has made efforts, such as making strategy changes to the control methods, launching the national carbon emissions trading market, and proposing plans to impose carbon tax [7,8,9]. China is now in a critical period of economic transition and cannot eliminate the country’s dependence on fossil energy in the short-term. How to realize the committed carbon emission reduction targets has been a key question in China for several years.

Carbon emissions refer to carbon dioxide emissions. Besides its natural formation, CO_2_ is mainly created by fossil energy combustion [10], especially combustion arising from industrial activities. Thus, in industrial production processes, an important question is whether the wastewater, exhaust gas and waste residue that are generated have effects on carbon emissions. To date, few studies have been reported which incorporate industrial pollution emissions into the carbon emission system [11,12]. If these two facets are related, there must be a logical connection. For one thing, industrial pollution emissions and carbon emissions have the same root: combustion of fossil fuel. In the combustion process, fossil energy can emit air pollutants such as sulfur dioxide, smoke and dust, as well as climate-harming pollutants such as CO_2_. Additionally, industrial pollution emissions are mainly manifested in wastewater, exhaust gas and solid waste residue. Accompanying the treatment of these waste streams is the release of a massive amount of CO_2_. For example, during aerobic treatment of wastewater to remove carbonaceous organic matter (OM), degradation of 1 kg of OM by microorganisms generates 2.02 kg of CO_2_ [13]. In 2004, an equivalent of 0.137 billion metric tons of CO_2_ in global greenhouse gas emissions were derived from the treatment of wastewater and solid waste residues [13]. The discharge of industrial wastewater is large, and the constitution of the wastewater is complex. Carbon dioxide emissions are also produced in the combustion process of industrial waste gas and industrial solid waste. It can be seen that the treatment of industrial pollution emissions will produce carbon dioxide emissions, which is inevitable. The close relationship between industrial pollution emissions and carbon emissions makes it necessary to incorporate industrial pollution emissions into the carbon emission system.

The interaction between industrial pollution emissions and carbon emissions can be considered from two aspects. One is the influence of industrial pollution emissions on carbon emissions, and the other is the influence of carbon emissions on industrial pollution emissions [14]. To provide a reference for carbon reduction targets, the study addressed the following problems: (1) whether industrial pollution emissions have pull effect on carbon emissions; (2) whether emission reduction can be promoted by the pull effect and, if so, in which regions; (3) over time whether the relationship between industrial pollution emission and carbon emission changes. The answers to these questions will provide reference for the identification of a time-space relationship between industrial pollution emissions and carbon emissions, as well as useful information for the realization of carbon reduction targets.

At present, only a few reports link industrial pollution emissions and carbon emissions, and few reports investigate the relationship between the two emissions. Against the background of emission reduction, most scholars have focused on identifying and analyzing driving factors and mechanisms influencing carbon emissions. In these studies, carbon emissions are mostly characterized by the total carbon emissions, per capita carbon emissions and carbon emissions per unit of gross domestic production (GDP) [15,16,17]. In China, the economic scale [18,19], energy mix [20], energy intensity [21], technical level [22,23], industrial structure [24,25] and opening of Chinese society to the outside world [26] serve as significant explanatory variables to investigate carbon emissions. Carbon emissions are mainly derived from the utilization of fossil energy. Due to the advantage of natural resources endowment, growth of the Chinese economy relies on the excessive consumption of fossil energy. The extensive production mode is limited by the technical level and economic condition of China’s industrial base, in which the energy use efficiency is low. The industrial structure is partial to heavy industrialization. All these characteristics of industry in China increase the complexity of the carbon emission issue. In theory, the application of technology can improve energy efficiency. However, the positive effect of technological factors on carbon emission reduction has not been verified comprehensively. If the technical level in an energy-consuming industry is low, capital investment, experimentally research and personnel management will increase the cost of technology and reduce the efficiency of energy utilization. Furthermore, there may be an energy rebound effect. A relatively high technical level that favors greater production causes higher energy consumption and generates more waste, both of which reduce the positive impact of improved energy use efficiency. For China, the opening of society to the outside world involves opening the local environment, demonstration and positive externality of foreign-funded enterprise, and the effects of these changes on carbon emissions have not been confirmed completely. In addition, the rate of renewable energy usage [27], urbanization [28], population size [29] and policy and regulation [30] have been shown to be common explanatory variables of carbon emissions.

In 2015, Chinese government proposed the “13th Five-Year Plan” [31], which represents the Chinese government’s plan for economic and social development in 2016–2020 [32], clarifying the focus of work, setting goals and indicating directions. In the “13th Five-Year Plan”, energy-saving and emission-reduction targets in China change from the simple target of total emission reduction to the dual targets of environmental quality improvement and controlling the total amount of pollutants. At present, the research on industrial pollution emissions is concentrated mainly on two aspects of this problem [33]. The first is the measurement of industrial pollution emission levels by weighted summation of various emissions [34]. The common methods for performing this calculation are the vertical and horizontal scatter degree method and the entropy weight method. The emphasis in both methods is to calculate the weights (i.e., proportional importance) of emissions from different sources. Alternatively, one or several typical pollutants (e.g., sulfur dioxide emissions, smoke and dust emissions, nitrogen oxide emissions, etc.) can be selected to represent the industrial pollution emission level [35]. Additionally, when measuring and monitoring industrial emissions, environmental performance index is employed by some institutions and scholars [36,37]. The second aspect of industrial emission research is the exploration of factors influencing industrial pollution emissions, including economic scale [38], technological factors [39], environmental regulation [40], energy intensity [41], ownership structure [42]. A review of existing literature indicates that the factors affecting industrial pollution emissions and carbon emissions have similar characteristics. To some extent, this similarity indicates that there exists a possible synergistic emission reduction strategy for both types of emissions.

China has a vast territory. Because the variables affecting carbon emissions exhibit the spatial heterogeneity characteristics, there exists great uncertainty in the research about carbon emission reduction [43]. To accurately reflect the degree to which each factor influences carbon emission, pertinent investigations at the regional level are more scientific and rational than those at a smaller or larger scale. The commonly used spatial econometric methods include spatial autocorrelation analysis (see Appendix A) [44,45], spatial lag models [46], spatial error models [47], geographically weighted regression models [48,49]. However, some spatial analysis methods are limited to the use of cross-section data or pay too much attention to spatially heterogeneous effects, while ignoring temporal fluctuations and spatial correlation. Furthermore, the effects of surrounding areas on a specific locality due to adjacent spatial locations are also neglected. Thus, the parameter regressions developed for a region can be biased to some extent.

A review of existing research shows that although the factors influencing the characterization of carbon emissions have been widely studied, the carbon emission system factors are complex. Furthermore, some variables that are not easy to observe have been omitted, and the effects of other variables on carbon emissions have been ignored, leading to the development of incomplete regression relationships. The economic developmental levels are imbalanced throughout China. Although different results can be obtained from the spatial econometric analysis method according to the differences in the actual situation among provinces, the time effect is not included, and the accuracy in the parameter estimation is reduced.

To address the inadequacies in existing research, this study extends it in the following ways: (1) in contrast to previous carbon emission indicators, calculations are made according to the carbon emissions per region. Since carbon emissions are characterized by carbon density, the effects of social factors are minimized as much as possible. In addition, using the vertical and horizontal scatter degree method, the weights of several typical pollutants are determined, making estimates of the industrial pollution emission level more comprehensive and accurate. The indicator system includes industrial sulfur dioxide emissions, industrial smoke (powder) dust emissions, industrial waste water emissions, production of industrial solid waste. (2) Industrial pollution emissions are incorporated into the carbon emission system. The relationship between the industrial pollution emissions and carbon density is also investigated. Moreover, it is examined whether there exists the possibility of synergistic emission reduction. In some literature, a synergistic emission reduction is assumed as the given condition, and on this basis the emission reduction is explored. But the possible influence on carbon emissions due to industrial pollution emission reduction activities is neglected. This study will address this gap. (3) The relationship between industrial pollution emissions and carbon emissions not only depends on current activities, but also may be affected by conditions in previous periods. When time is considered nonstationary and embedded into the econometric model, the accuracy of estimated results is improved, and the reliability is also reinforced. Moreover, as time passes, the spatial and temporal change characteristics of parameter estimation in different geographical units can be investigated. This provides more detailed information for policy makers. The remainder of this paper is divided as follows: Section 2 describes the methodology and data. Section 3 presents the results and analysis. Finally, we present the conclusions of this study.

## 2. Methodology and Data

### 2.1. Methodology

#### 2.1.1. Vertical and Horizontal Scatter Degree Method

The vertical and horizontal scatter degree method is mainly used to highlight the difference of the most distant research object. By establishing a time sequence three-dimensional data table, the weights are calculated, and the comprehensive index is obtained. The calculation process is as follows:

1. Data standardization:
  (1) $$x^{*}{}_{m,n,t}=(x_{m,n,t}-{\bar{x}}_{n,t})/\delta_{n,t}$$
  where m represents the province, and m = 1, 2, ..., 30. n is the index, and n = 1, 2, 3, and 4. t is the year, and t = 1997, 1998, ..., 2015. X*_m,n,t_ represents the value of a parameter after standardization. x_m,n,t_ is a “raw” datum before standardization. ${\bar{x}}_{n,t}$ is the mean value of n indexes in 30 provinces in year t. δ_n,t_ is the standard deviation of *n* indexes in 30 provinces in year t.
2. Calculation of the real symmetric matrix:
  (2) $$X_{t}=\left[\begin{array}{ccc} x^{*}{}_{1,1,t} & \cdots & x^{*}{}_{1,4,t}\\ \vdots & \ddots & \vdots \\ x^{*}{}_{30,1,t} & \cdots & x^{*}{}_{30,4,t} \end{array}\right]$$
  (3) $$H_{t}=X^{T}{}_{t}X_{t}$$
  (4) $$H=\sum_{t=1997}^{2015}H_{t}$$
  where H_t_ and H are the real symmetric matrices, and X^T^_t_ is the transpose matrix of X_t_.
3. Calculation of the comprehensive index:
  (5) $$X_{m,t}=2+\sum_{n=1}^{4}w_{n}\cdot x^{*}{}_{m,n,t}$$
  where X_m,t_ is the comprehensive index in province m in the year t. w_n_ is the weight, which is the normalization of the eigenvector corresponding to the maximum eigenvalue of H. x*_m,n,t_ is the value after the standardization. To facilitate the logarithmic processing of variable data, the calculation is magnified [50].

#### 2.1.2. Geographically and Temporally Weighted Regression Model

According to Appendix B, the ordinary least square (OLS) is a global regression model. It is assumed that the data acquired at many different geographical positions are equivalent to different data acquired at the same geographical position [51]. The geographical weighted regression (GWR) is the local regression model, which introduces the spatial attribute of a sample, but is only applicable to the cross-section data [52]. Based on GWR, the time effect is embedded in GTWR, which can reflect spatial and temporal change characteristics [53]. Thus, the reliability of a regression determined using GTWR is relatively high.

The core of GTWR is the space-time weight matrix. Based on the spatial weight of GWR, the time factor is added into the weight of GTWR. The spatial weight of GWR is calculated using the spatial weight function, most commonly the Gaussian kernel function. According to the setting of weight function method, the weight is relatively sensitive to the selection of band width. In this study, the optimal band width is determined using the cross-validation method. The fundamental process is described as follows:

The GTWR model is:

(6) $$y_{i}=\beta_{0}(u_{i},v_{i},t_{i})+\sum_{j=1}^{n}\beta_{j}(u_{i},v_{i},t_{i}^{})x_{ij}+\varepsilon_{i}$$

where i is the province, and i = 1, 2, ..., 30. j is the index, and j = 1, 2, ..., n. y_i_ and x_i_ are the explained variable and explanatory variable, respectively. β_0_ and β_j_ are the regression constant and regression parameter, respectively. (u_i_, v_i_) is the geographic coordinates, and (u_i_, v_i_, t_i_) is the space-time coordinates in *i*th province. ε_i_ is the residual term.

The space-time distance between the *i-*th and *j-*th province is:

(7) $$d_{ij}=\sqrt{\alpha \left[{\left(u_{i}-u_{j}\right)}^{2}+{\left(v_{i}-v_{j}\right)}^{2}\right]+\beta {\left(t_{i}-t_{j}\right)}^{2}}$$

According to the Gaussian kernel function, the weight of the sample point j relative to i is:

(8) $$w_{ij}=\exp(-d_{ij}{}^{2}/h^{2})$$

where, h is the band width, which is calculated using the cross-validation method, that is, the minimum sum-squared error rule:

(9) $$\min CV={\sum \left[y_{i}-{\hat{y}}_{\neq i}(h)\right]}^{2}$$

The space-time weight matrix is:

(10) $$W(u_{i},v_{i},t_{i})=diag(w_{i1},w_{i2},\ldots ,w_{in})$$

The parameter estimation using the GTWR model is:

(11) $$\hat{\beta }(u_{i},v_{i},t_{i})={\left[X^{T}W{\left(u_{i},v_{i},t_{i}\right)}^{2}X\right]}^{-1}X^{T}W{\left(u_{i},v_{i},t_{i}\right)}^{2}Y$$

### 2.2. Data Specification

In the study, carbon density is selected as the explained variable, and the comprehensive index of industrial pollution emissions is designated the core explanatory variable. After eliminating collinearity among data, the control variables are economic growth, opening to the outside world, industrial structure, technological advances and population density. Carbon dioxide emissions are calculated for three fossil energy sources (coal, petroleum and natural gas), and the calculation process is as follows:

(12) $$C=\sum_{i=1}^{3}\alpha_{i}\cdot E_{i}$$

where i = 1, 2, 3. C represents CO_2_ emissions. α_i_ is the carbon emission coefficient of the *i*th energy source. E_i_ is the consumption of the *i-*th energy source. The carbon emission coefficients for the three energy sources refer to the research of Hu and Huang [54] and Dong et al. [55], as well as reports from the Intergovernmental Panel on Climate Change [56].

To eliminate the effect of heteroscedasticity, a logarithmic transform is carried out for each variable, and the resulting model expression for CD is as follows:

(13) $$\ln CD_{it}=\alpha_{it}+\beta_{1it}\ln IPD+\beta_{2it}\ln GDPP+\beta_{3it}\ln OP+\beta_{4it}\ln IS+\beta_{5it}\ln TA+\beta_{6it}\ln PD+\varepsilon_{it}$$

where i is the province, and i = 1, 2, ..., 30. t is the year, and t = 1997, 1998, ..., 2015. α_it_ represents the intercept term, and β_it_ is the regression parameter of corresponding variable in the *i*th province. ε_it_ is the residual term. CD is carbon density. IPD is the comprehensive index of industrial pollution emissions. GDPP is economic growth. OP represents the opening to the outside world. IS represents the industrial structure. TA is technological advances, and PD is population density (see Table 1). Data (see Table 2) is from the China Statistical Yearbook and China Land Resources Yearbook.

### 2.3. Framework of the Research

**Figure 1:**
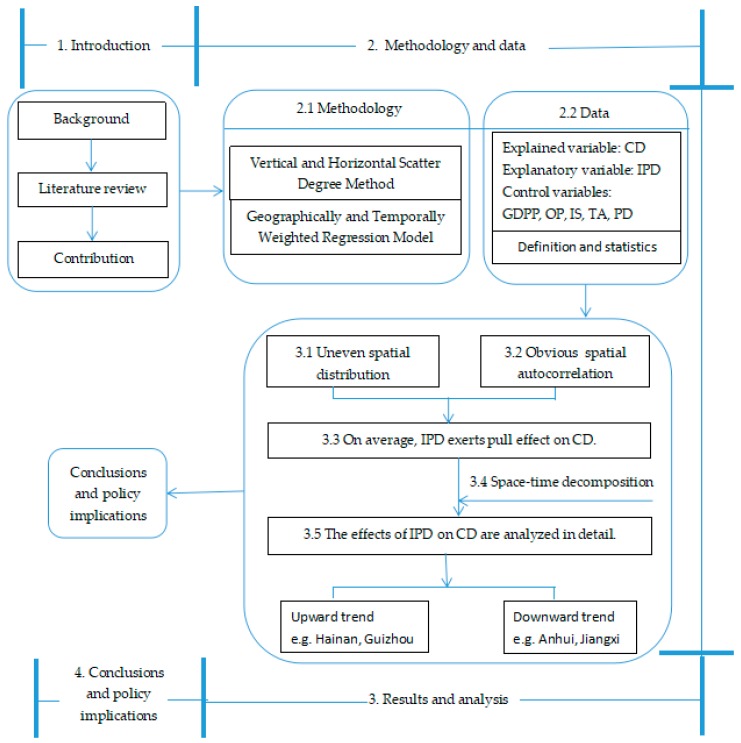
Framework of this research.

An overview of the research framework used in this study is shown in Figure 1. Against the background of carbon emission reduction, the role of industrial pollution emissions in carbon emissions is studied. Observing the spatial distribution characteristics of carbon density, it is found that with the advancing of time, obvious changes take place in different regions. Moran’s I indicates obvious spatial correlation of carbon density. The spatial correlation of industrial pollution emissions became increasingly obvious, justifying its inclusion as a variable in the GTWR model. Using the Herfindahl index, the heterogeneity of space and time is decomposed. The variations in the space-time effects of industrial pollution emissions on carbon density are observed. Finally, some pertinent suggestions based on the research results are developed.

## 3. Results and Analysis

### 3.1. Spatial Distribution Characteristics

**Figure 2:**
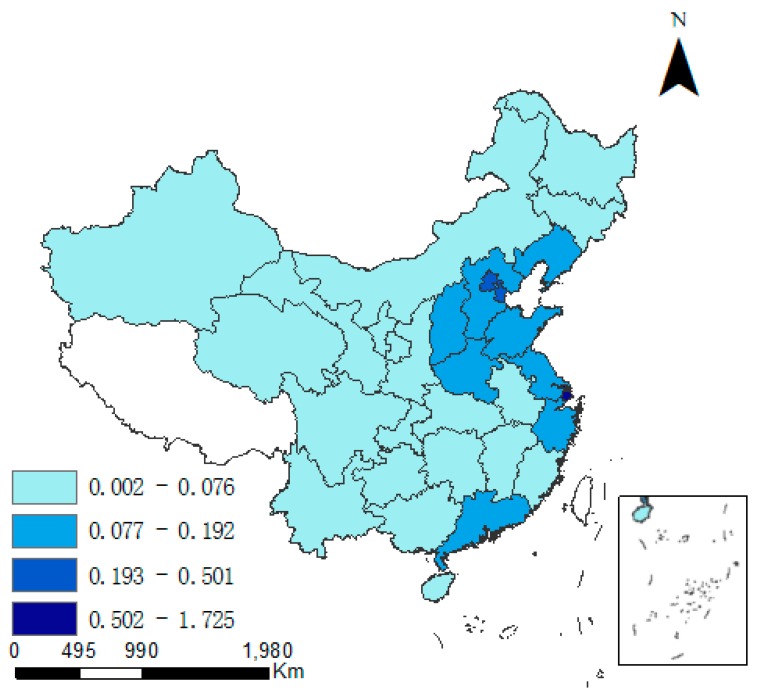
Carbon density in 1997.

**Figure 3:**
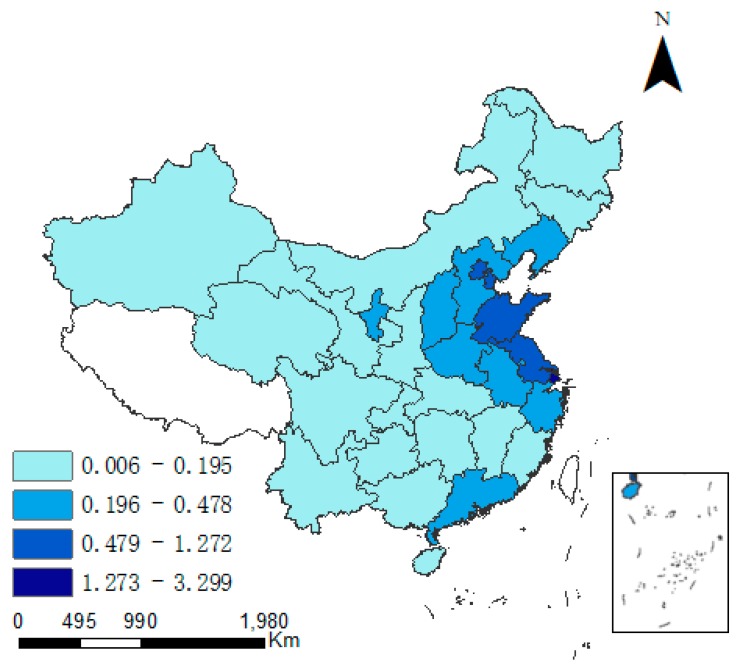
Carbon density in 2015.

There exist obvious spatial difference between CD and industrial pollution emission levels. As time progresses, variations in both indicators appear in some provinces. Figure 2 and Figure 3 specify the regional differences in CD (the unit is 10 K·t/Km^2^). In East China and North China, CD is was always higher than elsewhere. In 2015, CD was the highest in Shanghai (3.299) and Tianjin (1.272), but in all other provinces it was less than 1. However, the average annual growth rate in CD was the largest in the northwest region. In Ningxia, Xinjiang and Qinghai, the growth rate was consistently relatively high. During 1997–2015, the growth rate of CD was 12.9% in Ningxia (the highest nationwide), and was 9.9% and 7.7% in Xinjiang and Qinghai, respectively. In Shanghai and Beijing, the average annual growth rate of CD was 3.6% and 1.9%, respectively, which was the smallest in China.

**Figure 4:**
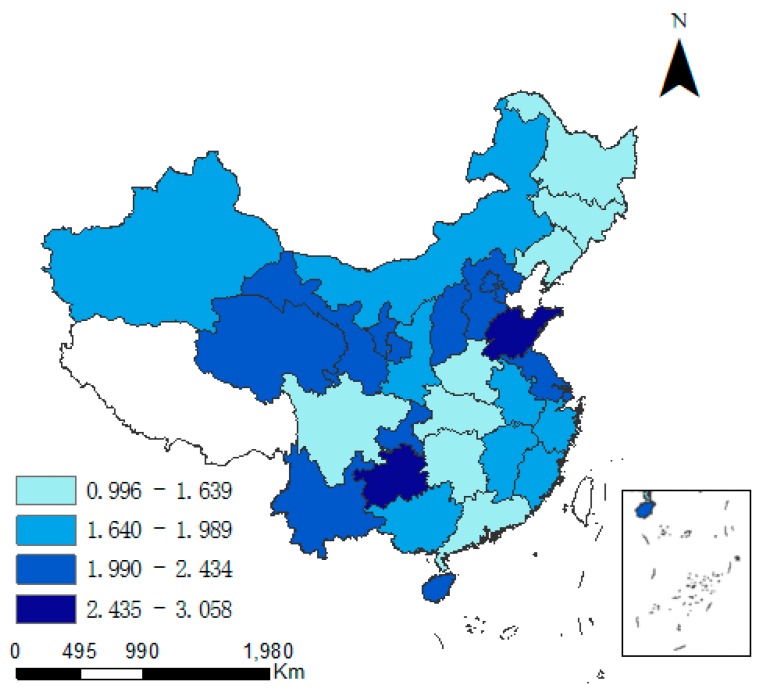
Industrial pollution emissions in 1997.

**Figure 5:**
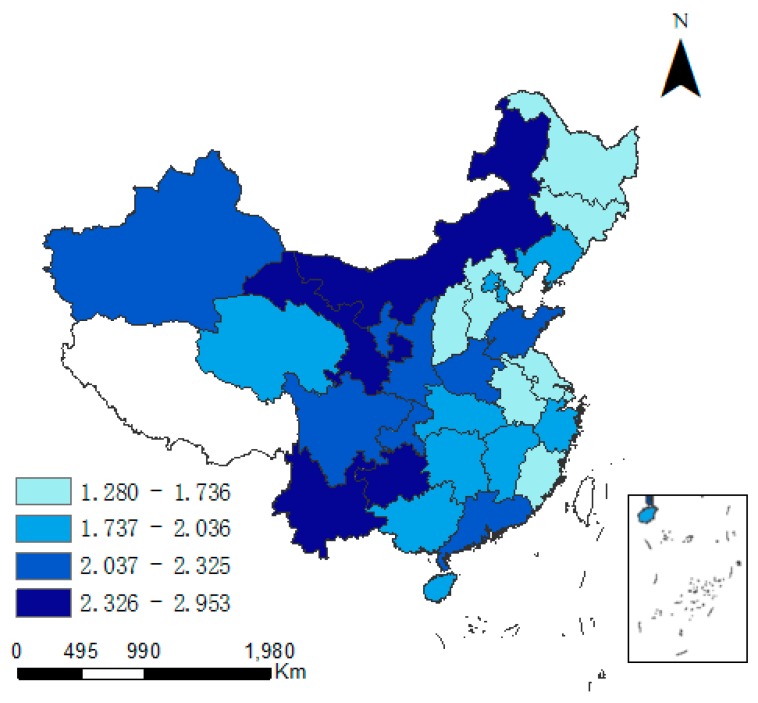
Industrial pollution emissions in 2015.

Figure 4 and Figure 5 show the regional differences in the industrial pollution emission levels. In most years, the industrial pollution emissions were the highest in Shandong. During 1997–2002, industrial pollution emissions declined in the most regions of Circum-Bohai-Sea Region and its radiation circle. However, there appeared to be a higher level of industrial pollution emissions in a portion of the western region from 2006.

In Sichuan, Xinjiang and Gansu, industrial pollution emissions were greater than 2, and were higher than the national average. The average growth rate in industrial pollution emissions was higher in the central and western regions than elsewhere. Henan exhibited the highest growth rate (5.4%), followed by that in Sichuan (4.7%) and Hunan (3.8%). The average growth rate in industrial pollution emissions in the eastern region was lower than in the central and western regions, and was negative in Shandong, Jiangsu and Shanghai.

### 3.2. Spatial Correlation Characteristics

**Figure 6:**
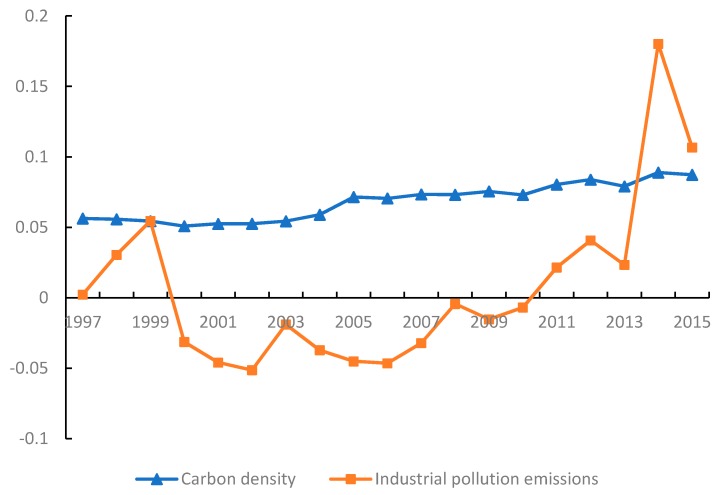
Moran’s I during 1997–2015.

Spatial correlation analysis is carried out for the CD and industrial pollution emissions during 1997–2015, and the Moran’s I of CD is positive. The z-values always exceed the critical value (1.96), indicating that at the 0.05 level of significance, there exists significant spatial autocorrelation for CD. Figure 6 shows that the values of Moran’s I for CD appear to be increasing, which suggests that the positive spatial autocorrelation is gradually reinforced over time. The changes in Moran’s I values for industrial pollution emissions present differences from those for CD, but all pass the z-test for significance at the 0.01 and 0.1 of levels in 2014 and 2015. Thus, industrial pollution emissions gradually exhibit positive spatial autocorrelation over time.

### 3.3. Analysis of Parameter Estimates

Based on the provincial panel data during 1997–2015, the goodness of fit of the model GTWR is 99.66%. The test of spatial autocorrelation on the residual of the regression shows that the residuals in all years are distributed randomly (see Table 3), indicating a strong regression and high reliability of the model.

Descriptive statistics of parameter estimates through GTWR model are presented in Table 4.

#### 3.3.1. Effect of Industrial Pollution Emissions on Carbon Intensity

The parameter estimates of LnIPD reflect the effect degree of industrial pollution emissions on CD. The positive values for the parameter estimates show that industrial pollution emissions exert pull effect on CD. In addition, CO_2_ can be generated in the governance process of industrial pollution emissions, and it deserves attention. Due to the existence of spatial-temporal heterogeneity, the estimated coefficients of the IPD variable specify different positive to negative characteristics.

#### 3.3.2. Effect of Economic Growth on Carbon Intensity

The parameter estimates of LnGDPP indicate the influence of economic growth on CD. The values of the parameter estimates for economic growth in each province are all positive, showing that economic growth increases CD. This is mainly because during the study period, China was (and still is) in a period of industrial development. Economic growth has not decoupled from energy consumption, and massive amounts of CO_2_ are released due to fossil energy combustion. Clean energy has not yet been widely used.

#### 3.3.3. Effect of Opening to the Outside World on Carbon Intensity

The parameter estimates of LnOP specify the effect of opening to the outside world on CD. In most provinces, the values are negative, showing that the opening to the outside world inhibits CD. In China, the introduction of advanced technology and new equipment improves China’s overall energy use efficiency. The technological advantages possessed by the foreign-funded enterprise play a role in positive externality. However, the “pollution heaven” phenomenon also exists in some regions where inefficient industry is concentrated. Thus, to some extent China’s opening of society to the outside world has a positive effect on CD in a few regions.

#### 3.3.4. Effect of Industrial Structure on Carbon Intensity

The parameter estimates of LnIS reflect the impact of industrial structure on CD. In most provinces, the values are positive. The secondary industry accounts for a comparatively larger proportion of the overall industrial structure. In China, the secondary industry includes four classes: mining; manufacturing; production and supply of electric power, fuel gas and water; and construction. Due to China’s endowment of natural resources, the secondary industry is dominated by extensive production techniques, and exacerbates the greenhouse gas emissions.

#### 3.3.5. Effect of Technological Advances on Carbon Intensity

The parameter estimates for LnTA show the effect of technological advances on CD. The values of the parameter estimates in each province are all positive. Although technological advances are beneficial to improving energy utilization efficiency, they can also give rise to a rebound effect. When technological advances develop to a certain extent, energy efficiency improvements can be overwhelmed by increases in energy consumption due to increased production capacity. Thus, the reduced energy consumption due to increased energy efficiency is offset, and technological advances promote (i.e., increase) carbon emissions.

#### 3.3.6. Effect of Population Density on Carbon Intensity

The parameter estimates for LnPD represent the influence of population density on CD. The values in each province are all positive. The population density is a common index to measure the degree of population agglomeration. Due to concentrated production activity, residential life and infrastructure development (e.g., construction of public utilities), the spatial form of a compact population can consume excessive amounts of energy. Additionally, an increase in the amount of residential and urban land in a given area compresses the planning space for land greening, which greatly reduces the opportunities to create carbon sinks.

### 3.4. Decomposition of Spatial-Temporal Heterogeneity for Parameter Estimates

Table 4 illustrates that the average effect of each variable on CD cannot show the space-time variation characteristics of parameter estimates. To more intuitively observe the spatial-temporal heterogeneity of parameter estimates, the heterogeneity in the time and space dimensions is decomposed using the Herfindahl index, so as to determine the dimension for selective analysis.

**Figure 7:**
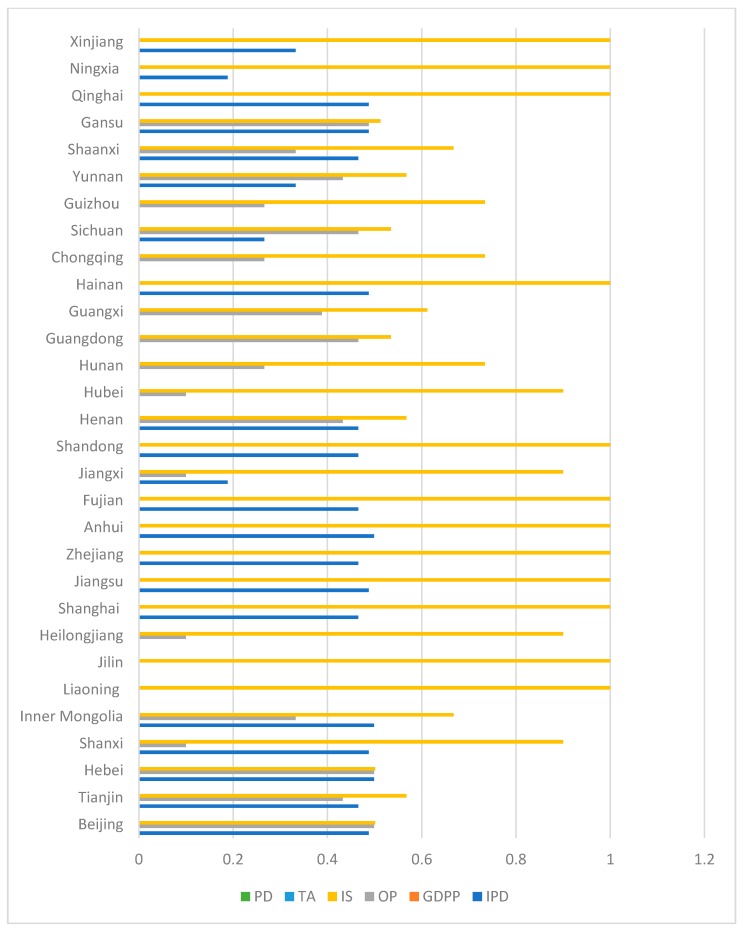
Temporal heterogeneity in spatial dimension.

**Figure 8:**
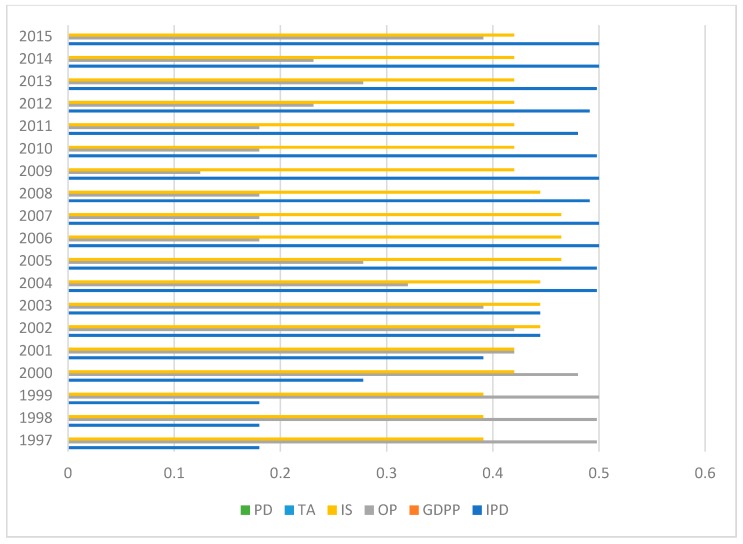
Spatial heterogeneity in time dimension.

In the Herfindahl index, H = 1 − (*p*^2^ + *q*^2^). *p* and *q* are the proportion of a parameter that is larger and smaller than zero, respectively. The larger H is, the greater is the imbalance [57]. Overall, spatial heterogeneity is more unstable than temporal heterogeneity (see Figure 7 and Figure 8), but each variable has different performance in different regions. Taking industrial pollution emissions as an example, the spatial heterogeneity in 2006, 2007 and 2009 is more obvious, and the temporal heterogeneity of Anhui, Hebei, Inner Mongolia and other places is more obvious. Therefore, it is necessary to add time factors from the local level for detailed analysis.

### 3.5. Spatial-Temporal Heterogeneity Analysis of Parameter Estimates

For convenience, taking the parameter estimates of industrial pollution emissions as an example, results are discussed according to the traditional division of eastern, central and western regions.

#### 3.5.1. Industrial Pollution Emissions and Carbon Density in Eastern China

**Figure 9:**
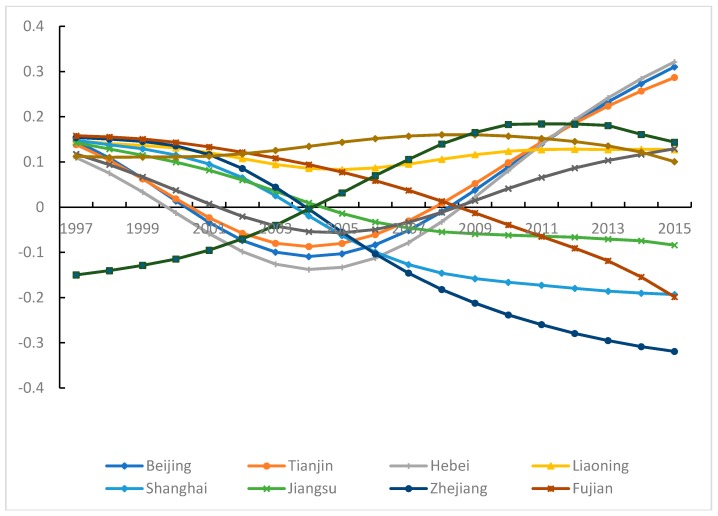
Parameter estimates of industrial pollution emissions in Eastern China.

In Beijing, Tianjin, Hebei and Shandong, there is a U-shaped curvilinear relationship between industrial pollution emissions and CD (see Figure 9). Especially during 2000–2008, the industrial pollution emissions did not stimulate an increase of CD. The main reason for this pattern is the undertaking and holding of the Beijing Olympic Games. Most of the industries with high pollution and high emission levels were transferred to Central and Western China. In addition, to promote environmental quality improvement, the technology for energy-saving and pollution treatment was widely applied, resulting in improved control of industrial pollution emissions. However, after 2008, industrial pollution emissions stimulated an increase of CD. This suggests that the controls on industrial pollution emissions and CO_2_ emissions were relaxed after the Olympic Games. A “backflow” of industry transfer also may have occurred.

In Shanghai, Jiangsu, Zhejiang and Fujian, the parameter estimates of industrial pollution emissions exhibit a continuous downtrend trend. This indicates that the pull effect of industrial pollution emissions on CD gradually weakens over time. Moreover, the pulling effect disappears around 2006. In recent years, in Shanghai, Jiangsu and Zhejiang, industrial structure has been constantly adjusted and upgraded, and gradually tended toward rationalization and high gradation. With the industry transfer, the high-level and light industrial structure will be realized quickly. Meanwhile, in the eastern region, the developmental level and energy efficiency are high. This region possesses advanced pollution treatment technology and has perfected pollution treatment instruments. In the pollution treatment process, lower carbon emissions can be realized. In Fujian, because of historical factors, constraints posed by a mountainous and hilly geographical environment and a poor economic foundation, in contrast to the other eastern provinces, there exist problems such as high proportion of the secondary industry and low technical efficiency. The pull effect of industrial pollution emissions on the carbon emissions is obvious. Until 2011, the pulling effect of industrial pollution emissions on the CD gradually weakens. In 2015, there appears a downtrend trend in the proportion of secondary industry. In 2018, Fujian proposed that pollution control should be promoted. Therefore, the pulling effect of industrial pollution emissions on the CD would become weaker and weaker.

In Guangdong and Liaoning, the parameter estimates of industrial pollution emissions are always positive, and the fluctuation range is smaller than in other areas. In 1997, the industrial pollution emission level in Guangdong ranked 23rd nationwide. In 2015, it ranked 7th, and was above the national average. During 1997–2015, the industrial pollution emission level increased 33%, and the rate of increase ranked 5th nationwide. The pull effect of increased industrial pollution emissions on CD is obvious. Meanwhile, the rebound effect caused by the technological advance impacts the previously reduced energy consumption that was achieved through energy efficiency improvement. Liaoning is a key province in the initial period of industrial development. The proportion of the secondary industry is high, and the industrial pollution emissions are always at a relatively high level. There exists an accumulative effect for pollution. In the state-owned enterprises, the problems such as solidified structures, rigid management systems and slow development gradually appear. The government protection for state-owned enterprise results in the softening of environmental constraints. Additionally, within the Northeast Revitalization Plan, the development speed of Liaoning is the lowest among the three provinces. The improved effect of management techniques on environmental quality is not apparent.

In Hainan, the parameter estimates of industrial pollution emissions increase significantly. After 2004, industrial pollution emissions have a positive effect on CD. In Hainan, although the leading industries are tourism and services, the proportion of secondary industry has been increasing. In addition, the proportion of heavy industry in industrial structure is increasing. In 2011, heavy industry was three times larger than light industry. The industrial pollution emission level has gradually approached the national average. In the short term, there may be continuous pull effect of industrial pollution emissions on CD.

#### 3.5.2. Industrial Pollution Emissions and Carbon Density in Central China 

**Figure 10:**
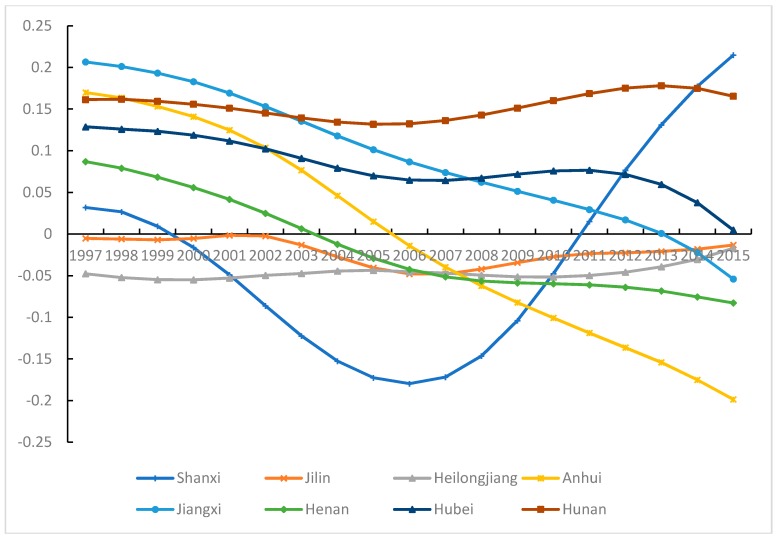
Parameter estimates of industrial pollution emissions in Central China.

In Shanxi, there is a U-shaped curvilinear relationship between industrial pollution emissions and CD (see Figure 10). The effect declines sharply from 1999, then reaches a minimum in 2006, and rises continuously thereafter. Starting from the bid for the Olympic Games in 1999, Shanxi began to reinforce the supervision to industrial enterprise and to control pollution emissions. In 2006, the policy “Control Method for Major Industrial Pollution Sources in Shanxi Province” was passed, which targets heavily polluting enterprises. However, since the 2008 Olympic Games, pollution control has been relaxed. In addition, Shanxi is a natural resource-dependent province and industrial pollution emissions are substantial. Medium and small enterprises (especially) face problems such as insufficient funds for pollution treatment, immature treatment techniques and imperfect equipment. In the pollution treatment process, an enormous amount of CO_2_ is generated. In the pursuit of GDP growth, there exist problems such as failed environmental regulation in most of the region, aggravating the pull effect of industrial pollution emissions on CD.

In Jilin, Heilongjiang and Hunan, the parameter estimates of industrial pollution emissions fluctuate less than in other areas of the region. The values are negative for most years in Jilin and Heilongjiang, and industrial pollution emissions did not promote CD. Jilin and Heilongjiang experience problems similar to those in Liaoning, such as lagging industrial development, an unsound industrial system and ageing equipment. The two provinces have grasped the strategic opportunity provided by the “Northeast Area Revitalization Plan” to optimize industrial structure, strengthen economic cooperation with foreign countries, and promote innovation ability. Due to natural geographic conditions, some pollution in Jilin and Heilongjiang is transported to Liaoning, decreasing the apparent level of pollution in the two provinces. In Hunan, industrial pollution emissions have a pull effect on CD. In the process of transferring industries to the mainland in the eastern region, Hunan has a regional development advantage in connecting east and west, as well as north and south, and especially for joining with Guangdong. During 1997–2005, the proportion of the secondary industry increased by 5.3%. However, when Hunan accepts new transferring industry, it also accepts the resulting pollution emissions. Economic growth in Hunan has exhibited extensive characteristics, and its high population density promotes CO_2_ emissions.

In Anhui, Jiangxi, Henan and Hubei, the parameter estimates of industrial pollution emissions are decreasing over time. After 2008, the industrial pollution emissions does not show pull effect on CD in Anhui and Henan, while this phenomenon appears in Jiangxi and Hubei only in the recent few years. Anhui and Henan are the major agricultural provinces in China; consequently, the proportion of industry in these provinces is relatively low. After implementation of the “Rise of Central China Plan”, the industrial economy developed rapidly in Jiangxi and Hubei and energy consumption increased, as did the industrial pollution emissions and carbon emissions. With this economic transition and adjustment of industrial structure, the industrial economy has been reinforced, and energy saving and emission reduction technology has been applied positively, improving the energy utilization efficiency. The effect of industrial pollution emissions on CD is weakened.

#### 3.5.3. Industrial Pollution Emissions and Carbon Density in Western China

**Figure 11:**
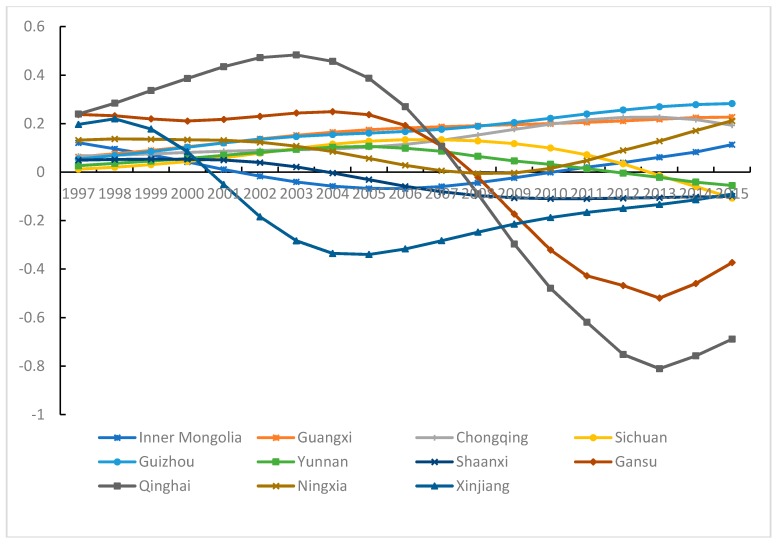
Parameter estimates of industrial pollution emissions in Western China.

There appears an increasing trend between industrial pollution emissions and CD in Inner Mongolia, Guangxi, Chongqing, Ningxia and Guizhou (see Figure 11). In Inner Mongolia, the relationship has become more strongly positive, but with fluctuation. After 2010, the pull effect of industrial pollution emissions on CD has been reinforced. In 2000, Inner Mongolia was included within the scope of China’s Western Development Plan. As a natural resource-oriented province, its industrial structure is biased toward heavy industrialization.

During 1997–2015, industrial pollution emissions increased by 61.86%, and the rate of increase was the highest in the country. In the short term, energy mix oriented toward coal cannot be changed, and the pull effect on CD will become more obvious. In Guangxi, Chongqing and Ningxia, the increasing trend between industrial pollution emissions and CD is small. Guangxi is in a period of rapid development and environmental pollution is accompanying economic growth. Guangxi accepts high-investment, high-emission and high-pollution industry in the eastern and central regions, especially in Guangdong. Recently, although the industrial pollution emissions have declined, the governance of industrial pollution emission has not advanced in Chongqing and Ningxia. The pull effect of industrial pollution emissions on CD is relatively strong in Guizhou because energy mix is based mainly on raw coal. In 2010, the “industrialized province” strategy was launched. The pulling effect of industrial pollution emissions on CD is gradually reinforced.

In Sichuan and Yunnan, the relationship between industrial pollution emissions and CD initially increases but then decreases during the study period. The southwest region has one of the richest mines in China. Since implementation of the Western Development Strategy in 2000, the industrializing economy has developed quickly. The industrial pollution emissions have increased significantly, and the pull effect on CD has become gradually stronger. In 2007, there appeared the industry transfer phenomenon. Due to the advantages of low factor cost and great market potential, the western region accepts some industries from the eastern and central regions. In recent years, the industry transfer has given attention to both pollution control and treatment simultaneously. Consequently, the effect of industrial pollution emissions on CD has weakened.

In Gansu and Qinghai, the parameter estimates of industrial pollution emissions decrease on the whole, and in Shaanxi, the decrease is slight. In Gansu, a U-shaped curvilinear relationship between industrial pollution emissions and CD develops after 2006. The industrial pollution emission level is high in Gansu, particularly in Lanzhou. Due to the local terrain and relatively weak air convection, industrial waste gas emissions have a stronger effect on CD here. After a pollution accident in 2004, pollution control has gained increased importance. In the process of accepting industry transfer, more attention has been placed on upgrading economic structure and applying high technology and new technology. Thus, industrial pollution emissions did not promote CD after 2008. In Qinghai, an N-shaped curvilinear relationship characterizes the effect of industrial pollution emissions on CD. The ecological environment in Qinghai is fragile, and over the years, environmental protection has always been greatly important. Due to the China Western Development plan, the number of industrial enterprises has increased, as has the level of industrial pollution. Because of economic restrictions and inadequate control techniques, the industrial pollution emissions promoted CD. After the industry transfers became more numerous and Qinghai became more industrialized, new technology was applied positively and pollution treatment shows an obvious beneficial effect. Shaanxi has received major benefits from technological advances; as a result, the energy utilization efficiency and pollution treatment efficiency have improved, so that industrial pollution emissions have no pulling effect on CD.

In Xinjiang, a U-shaped curvilinear relationship exists between industrial pollution emissions and CD. The values are negative from 2001 and trend tends to be stable after 2011. The industrial pollution emission level increased continuously in Xinjiang. Due to the terrain surrounded by mountains, pollutant diffusion is restricted. However, as Xinjiang is located along the border of the northwest region, and positively advances opening itself to the outside world, introduces techniques and new equipment, foreign-funded enterprises also have had a demonstrable effect. The governance of industrial pollution emissions has also gradually improved. In 2014, the proportion secondary industry started to decline. Thus, industrial pollution emissions did not promote CD.

## 4. Conclusions and Policy Implications

This paper applied a GTWR model to study the relationship between industrial pollution emissions and carbon emissions, used the Herfindahl index to decompose space-time heterogeneity, and then analyzed the situation in various regions at the time and space level. The results show that the regression fit of GTWR model is high, therefore, the results are significant and reliable. The spatio-temporal heterogeneity has different degrees of impact on different regions at different times, therefore, different regions need to be analyzed according to different situations.

Overall, the average effect of industrial pollution emissions on carbon density is positive, indicating that industrial pollution emissions exert a pull effect on carbon emissions. It specifies that it is possible to achieve both environmental quality improvement and emission reduction targets. Besides China’s opening to the outside world, several other control variables (economic growth, industrial structure, technological advances and population density) increase carbon density.

From the regional perspective, in Guizhou, Guangxi and Hunan, industrial pollution emissions have pull effect on carbon density, and carbon emission reduction could be promoted through industrial pollution emission control. However, in Shanghai and Shaanxi, the effect of industrial pollution emissions on carbon density is negative, environmental quality improvement and carbon reduction targets in these areas may not be achieved simultaneously.

By observing the variation trend of the parameter estimates, it is found that the industrial pollution emission coefficient in some regions has an upward trend, such as Hainan. It indicates that the pull effect of industrial pollution emissions on carbon density is increasing. However, the industrial pollution emission coefficient of some regions shows a downward trend, such as Anhui and Jiangxi. It demonstrates that the pull effect of industrial pollution emissions on carbon density is weakening or disappearing.

In China, great differences exist in the natural and social conditions across the country, and the economic development level in 30 provinces is vastly different. It is necessary to fully take the spatial and temporal heterogeneity into consideration and implement a differentiated carbon emission strategy. To develop and implement a differentiated strategy, the following suggestions are proposed.

(1) Adjust energy mix and change development mode

In the short term, energy supply and consumption dominated by coal cannot be changed. Thus, industrial pollution emissions might increase continuously. For long-term reductions in industrial pollution emissions, it is necessary to positively adjust the industrial energy consumption structure, decrease the proportion of fossil energy consumption. Industrial pollution emissions from resource provinces such as Shanxi and Inner Mongolia have a significant pull effect on carbon density. We should actively optimize energy mix, apply new technologies to improve energy efficiency, and minimize carbon dioxide emissions in the treatment of industrial pollutants. As problems such as structural consolidation and difficulty in transforming state-owned enterprises have arisen in Liaoning, exchange of experience with other regions should be strengthened. Shanghai, Zhejiang and other regions with high economic level should build high-tech demonstration parks, strengthening itself while guiding and demonstrating the central and western regions.

(2) Promote industry transfer and control the flow of pollution

In China, industry transfer mainly manifests as the transfer of “three high” industries (those that have high pollution, high energy consumption and high emission). In the eastern region, international industries should be selectively accepted, so as to prevent the region from becoming a “pollution heaven” for inefficient and highly polluting enterprises. Likewise, in some areas such as Hebei and Beijing, care must be given to assure that domestic industry transfer does not cause a “backflow” of pollution. Because the central region is a crossroad in domestic industry transfer, during the advancement of these industry transfers, all regions should seize the opportunity to eliminate the use of outdated equipment, break the original property-right structural pattern and formulate reasonable industry development plans, such as Shanxi. So is Liaoning in the east. The western region should grasp the opportunity of industrial upgrading, reduce the repeated transfer of highly polluting industry as far as possible, and avoid the old way of “pollution first, treatment later”. Areas such as Qinghai and Yunnan with fragile ecology must prevent the ecological deterioration.

(3) Introduce advanced equipment and adopt high technology and new technology

In most Chinese provinces, the opening of society to the outside world inhibits carbon density. Thus, especially for Liaoning, it is necessary to advance the opening to the outside world continuously, encourage indigenous industrial enterprises to learn from advanced international experience and to adopt advanced technology for pollution treatment. Technological advances often cause an “energy rebound effect”, thus, other supplementary means should be used, for example, strengthening the cooperation among low-carbon and low-pollution projects and increasing the quality of industry transfer that occurs due to opening to the outside world, such as Guangdong. In each province, the evolution path for technological advances must be analyzed. Provinces should avoid the increase in energy consumption due to the “energy rebound effect” of advanced technology, actively innovate and improve pollution treatment technology, and continuously strive to control pollution emissions.

(4) Strengthen the legal system and play a role of government

Through its administrative powers, the government should audit industries strictly, especially as regards project construction and factory establishment. Regulatory oversight also should be exercised over operations in the heavy chemical industry. A mobile supervision group can be established, and the frequency and proportion of spot tests should be increased. For industrial enterprises that do not treat pollution discharges as required and remediate problems in a timely manner, public disclosure can be used to highlight the unacceptable environmental behavior. Equally, it is vital to encourage enterprises that have low emission levels and utilize positive pollution treatment techniques. In the eastern region, such as Beijing, a “backflow pollution” phenomenon exists, and the government should play a strict regulatory role in correcting this problem. The central region, such as Shanxi, is to reinforce the legally binding environmental controls on enterprises, and strictly control the pollution emissions. Provinces in the western region, such as Qinghai and Yunnan, must address the dual tasks of economic development and ecological protection. It is necessary for the government to adopt a support policy and accelerate the pace of economic development in this region by injecting capital.

## Figures and Tables

**Table 1:** Definition of all variables.

Variable	Definition	Variable	Definition
Carbon density (CD)	Carbon dioxide emissions/administrative land area (10 K·t/Km^2^)	Industrial structure (IS)	Second industry added value/GDP (%)
Industrial pollution emissions (IPD)	Comprehensive index	Technological advances (TA)	Energy consumption/GDP (t/10 K·yuan)
Economic growth (GDPP)	GDP/total population (yuan)	Population density (PD)	Total population/administrative land area (ren/Km^2^)
Opening to the outside world (OP)	Total imports and exports/GDP (%)		

**Table 2:** Statistical description of all variables.

Variable	Mean	Median	Max	Min	Std. Dev.
Carbon density	0.261	0.113	3.544	0.002	0.514
Industrial pollution emissions	2.000	1.986	3.175	0.570	0.405
Economic growth	17,169.490	13,393.418	69,336.200	2195.508	12,857.210
Opening to the outside world	0.330	0.126	12.806	0.032	0.653
Industrial structure	46.470	47.700	61.500	19.700	7.599
Technological advances	1.755	1.519	5.088	0.510	0.900
Population density	0.041	0.027	0.383	0.001	0.057

**Table 3:** Spatial autocorrelation test of the residual.

Year	Z-Test	Year	Z-Test	Year	Z-Test	Year	Z-Test
1997	0.629 *	2002	0.542 *	2007	0.252 *	2012	−0.600 *
1998	−0.463 *	2003	−0.665 *	2008	−0.707 *	2013	−0.164 *
1999	−0.663 *	2004	−0.042 *	2009	−0.397 *	2014	0.118 *
2000	0.061 *	2005	1.651 **	2010	2.385 ***	2015	1.930 **
2001	0.821 *	2006	0.494 *	2011	−0.310 *		

Note: *, **, *** denote 10%, 5%, 1% levels of significance, respectively.

**Table 4:** The statistical description of all parameter estimates (Band width = 0.115).

Index	Min	1/4 Quantile	Median	3/4 Quantile	Max	Mean
LnIPD	−0.811	−0.049	0.062	0.134	0.483	0.035
LnGDPP	0.637	0.892	0.946	1.031	1.398	0.960
LnOP	−0.316	−0.070	−0.030	−0.003	0.208	−0.036
LnIS	−0.955	−0.120	0.298	0.479	1.388	0.193
LnTA	0.639	0.975	1.098	1.236	1.740	1.102
LnPD	0.657	0.931	1.063	1.125	2.329	1.062

## Appendix A

The spatial correlation analysis is the first step of spatial analysis. According to Tobler’s First Law of Geography, there exists a closer relationship between adjacent objects than between objects separated by some distance, i.e., spatial autocorrelation. The common method to test for spatial autocorrelation is Moran’s index method (Moran’s I) [58]. The equation for calculating Moran’s I is:

(A1) $$I=\frac{\sum_{i=1}^{n}\sum_{j=1}^{n}\left(x_{i}-\bar{x}\right)(x_{j}-\bar{x})}{S^{2}\sum_{i=1}^{n}\sum_{j=1}^{n}w_{ij}}$$

(A2) $$S^{2}={\sum_{i=1}^{n}\left(x_{i}-\bar{x}\right)}^{2}/n$$

where i and j denote province, and both i and j = 1, 2, ..., 30. x_i_ and x_j_ are the carbon density in ith and jth province, respectively. $\bar{x}$ is the mean carbon density. w_ij_ is the spatial weight matrix. To better explain the relationships among adjacent regions, Rook’s spatial weight matrix is applied, which is expressed as follows:

(A3) $$w_{ij}=\left\{ \begin{array}{l} 1,\;when\;ith\;province\;is\;adjacent\;to\;jth\;province\\ 0,\;when\;ith\;province\;is\;not\;adjacent\;to\;jth\;province \end{array}\right.$$

The Z-test is carried out on Moran’s I values. In general, at the 0.05 significance level, when *z* is larger than 1.96, there exists positive spatial autocorrelation. When *z* is smaller than −1.96, there exists negative spatial autocorrelation. When *z* is in the range of [−1.96,1.96], no spatial autocorrelation exists.

## Appendix B

The OLS model is:

(A4) $$y_{i}=\beta_{0}+\sum_{j=1}^{n}\beta_{j}x_{ij}+\varepsilon_{i}$$

The GWR model is:

(A5) $$y_{i}=\beta_{0}(u_{i},v_{i})+\sum_{j=1}^{n}\beta_{j}(u_{i},v_{i})x_{ij}+\varepsilon_{i}$$

The spatial distance between the ith and jth province in GWR is:

(A6) $$d^{\prime }{}_{ij}=\sqrt{(u_{i}-u_{j}{)}^{2}+(v_{i}-v_{j}{)}^{2}}$$
